# The Effect of Secondary Education Teachers’ Metacognitive Knowledge and Professional Development on Their Tacit Knowledge Strategies

**DOI:** 10.3390/jintelligence11090179

**Published:** 2023-09-06

**Authors:** Maria Sofologi, Evaggelia Foutsitzi, Aphrodite Papantoniou, Georgios Kougioumtzis, Harilaos Zaragas, Magdalini Tsolaki, Despina Moraitou, Georgia Papantoniou

**Affiliations:** 1 Laboratory of Psychology, Department of Early Childhood Education, School of Education, University of Ioannina, 451 10 Ioannina, Greece; efoutsitzi@gmail.com (E.F.); afropapanto@gmail.com (A.P.); gpapanto@uoi.gr (G.P.); 2 Institute of Humanities and Social Sciences, University Research Centre of Ioannina (U.R.C.I.), 451 10 Ioannina, Greece; 3 Department of Early Childhood Education, School of Education, University of Ioannina, 451 10 Ioannina, Greece; hzaragas@gmail.com; 4 Department of Turkish and Modern Asian Studies, National and Kapodistrian University of Athens, 157 72 Athens, Greece; georgetype@gmail.com; 5 Laboratory of Neurodegenerative Diseases, Center of Interdisciplinary Research and Innovation (CIRI—AUTH), Balcan Center, Buildings A & B, 10th km Thessaloniki-Thermi, 541 24 Thessaloniki, Greece; tsolakim1@gmail.com (M.T.); demorait@psy.auth.gr (D.M.); 6 School of Medicine, Aristotle University of Thessaloniki, 541 24 Thessaloniki, Greece; 7 Greek Association of Alzheimer’s Disease and Related Disorders (GAADRD), 546 43 Thessaloniki, Greece; 8 Laboratory of Psychology, Department of Cognition, Brain and Behavior, School of Psychology, Aristotle University of Thessaloniki, 541 24 Thessaloniki, Greece

**Keywords:** practical intelligence, professional growth, high school teachers

## Abstract

The present study investigated the pattern of relations among the tacit knowledge of high school teachers, their professional development, and their metacognitive knowledge concerning their teaching practices. Two hundred and seventy-nine secondary school teachers of both sexes, between the ages of 30 and 59 years, with teaching experience of between 1 and 19 years, participated in the study. Teachers’ tacit knowledge was evaluated through the hypothetical scenarios of the Tacit Knowledge Inventory for High School Teachers (TKI-HS), which has been developed for the estimation of teachers’ practical strategies. For the evaluation of teachers’ metacognitive knowledge and professional development, self-report questionnaires were administered to the participants. Path analysis indicated relationships between teachers’ metacognitive knowledge regarding difficulties in classroom management and in the use of modern methods and technologies on the one hand, and the use of certain tacit knowledge strategies on the other. In addition, teachers’ professional development, especially their ability to interact in socially heterogeneous groups, was also found to have an effect on their tacit knowledge strategies.

## 1. Introduction

### 1.1. Tacit Knowledge

The philosopher Polanyi (1966) was the first to discuss and develop the concept of tacit knowledge. In specific, according to Polanyi, the first kind of knowledge is explicit knowledge. Explicit knowledge is expressed through words, numbers, diagrams, and other symbols. It is personal knowledge that has been formally and methodically organized or codified so that it can be transmitted. Tacit knowledge is the second type of information according to the philosopher. Since it is subjective and challenging to standardize, tacit knowledge cannot be clearly defined, making it challenging to express. In his view, individuals can know more than they can tell, i.e., they have knowledge that they cannot easily make explicit. Each type of knowledge, according to Polanyi, is personal, and he supports the idea that all forms of knowledge eventually lead to action. Furthermore, he argues that for tacit knowledge to be transmitted, the individual must first become aware of that knowledge and then find a way to express that knowledge (Matthew and Sternberg 2009). On the other hand, tacit knowledge is seen by Sternberg (1997) as condition–action planning or action-oriented. This approach incorporates both Schön’s emphasis on action and the effects of action as experienced via experience and Polanyi’s emphasis on stimuli or conditions from which an experience is developed (Matthew and Sternberg 2009). More specifically, in the context of the theory of intelligence for success, Sternberg and colleagues (Sternberg 1997; Sternberg and Hedlund 2002; Sternberg et al. 2001) defined tacit knowledge as an expression of practical intelligence, as the knowledge that reflects the practical ability to learn from experience and use that knowledge to achieve goals of personal value. More specifically, tacit knowledge, which is deeply rooted in action and context, can be acquired without awareness. In contrast, explicit knowledge is that which is articulated, codified, and transmittable through formal, systematic language (Sternberg 1997; Sternberg et al. 2001). Analogously, action-oriented knowledge or tacit knowledge is characterized by three different traits according to Sternberg (Sternberg et al. 1995) which concern (a) its structure, (b) the conditions under which it can be acquired, and (c) the conditions in which it can be used (Sternberg et al. 2001). It is the kind of knowledge that categorizes people into more and less practically successful in their workplace and is acquired while performing their daily activities without consciously knowing what they are learning. And while their actions reflect their level of knowledge, they often find it very difficult to verbally articulate or communicate what they know. In terms of a work environment, the knowledge to manage one’s time, one’s tasks, or to manage others represents different facets of tacit knowledge (Sternberg et al. 1995). As a result, aligned with knowledge management is experience-based learning or tacit knowledge, which can be viewed as a necessary condition for the acquisition of these skills since what the individual learns from oriented-based experience plays a significant role in the learning process (Matthew and Sternberg 2009). The individual is not directly instructed as to what they should learn but rather they must extract the important lesson from the experience, even when learning is not the primary objective. Consequently, the practical ability to learn from experience involves and activates cognitive processes through which tacit knowledge is applied to the way of solving practical problems in new experiences and amended by these new experiences (Matthew and Sternberg 2009; Saw and Han 2022). Additionally, tacit knowledge is related to goal achievement, an action that has great personal significance for each person individually. The higher a goal is, the more directly the achievement of that specific goal rests on knowledge and the more useful knowledge is (Foutsitzi et al. 2020). Furthermore, tacit knowledge acquisition is not achieved by environmental support. The concept of environmental support refers either to people or to those means that can help the individual gain knowledge and enhance the learning process (goal-oriented assistance in distinguishing or filtering more or less useful information, suggestions in synthesizing elements of information in useful ways, encouragement to compare the current situation with analogous background knowledge) (Elliott et al. 2011; Grigorenko et al. 2006). In other words, tacit knowledge is the result of personal effort since the individual alone must draw the conclusion about what is important to learn in each specific situation, always based on their own experience, even if learning is not what is required in a particular situation or field of specialism (Saw and Han 2022). Research data support the existence of a relationship between experience and tacit knowledge. The correlation found, however, is modest, showing a range of values between 0.20 and 0.40 (Sternberg et al. 2001; Grigorenko et al. 2006) and suggesting that tacit knowledge arises only partially from experience without registering an absolute correlation between them. Consequently, a person’s experience in a professional field cannot be considered representative of the acquired tacit knowledge. Two people may work for the same amount of time in a workplace. However, time is not a key factor associated with tacit knowledge. The most decisive factor in obtaining it is the way in which a person manages the experiences they gain in their professional environment. The development of expertise, regardless of the professional field, results from a process in which a maximum and conscious effort is made, which favors the accumulation of tacit knowledge that can be easily stored effectively and used to recognize analogies in the conditions that arise, enabling fast and effective decision making (Sternberg et al. 2001; Foutsitzi et al. 2020; Stemler et al. 2006; Scully et al. 2019).

### 1.2. Teachers’ Professional Development/Growth

In educational settings, tacit knowledge has been shown to be related to professional effectiveness (Stemler et al. 2006), as it has for many other professional fields requiring a high level of specialization. Researchers claim that it will be useful for teachers to receive more structured and systematic training on potential strategies for dealing with the various social situations they encounter daily to develop their tacit knowledge, which would help them improve their expertise (Scully et al. 2019; Malm and Löfgrer 2006). A review of the literature reveals that teachers’ efficiency and competence often highlight subtly differing parameters like subject knowledge (expertise in the subject being taught), pedagogical knowledge (expertise in approaches to teaching and learning), and a third that is concerned with a variety of professional characteristics (including positive relationships) (Malm and Löfgrer 2006; Elliott 2009). For many researchers, professional development is perceived as a systematic process of teacher improvement, which facilitates educators to change attitudes, beliefs, and practices to improve their students’ performance (Elliott 2009). In particular, Vescio et al. (2008) identified professional development as the development of an individual in their professional role and pointed out that professional development significantly influences teachers’ beliefs and practice, students’ learning process, and the implementation of reform changes. Through the effort to conceptualize professional development, several factors are highlighted to influence its formation (continuous learning and updating on issues related to the professionalism of teachers) (Bredeson 2000), critical thinking (Darling-Hammond 1999), and quality of teaching (Smith et al. 2007). In parallel, Avalos (2011) points out that the focus of many theoretical approaches to determine and define teacher professional development, regardless of their differences, focuses on the view that professional development is about teachers’ learning, as well as the way they learn and transmit their knowledge to their students, improving their learning and contributing to their development.

On the other hand, assisting and mentoring teachers to cope with the challenges inherent in the interpersonal domain of school life is a demanding objective as it is quite difficult for educators to provide their knowledge to their students as much of it is tacit. This makes it hard to be made explicit as a set of guiding rules for action (Sternberg 1997; Avalos 2011). In addition, teachers are often unaware of the skills they bring to bear in relation to the management of difficult interpersonal encounters, so describing or analyzing this implicit knowledge is almost impossible (Scully et al. 2019). Consequently, improving teacher training is an essential parameter for improving the quality and effectiveness of education. Teachers’ professional development is a key factor in the quality of educational work as it significantly impacts teachers’ beliefs, behavior, and practices (Darling-Hammond 1999; Sternberg 2005; Jennings and Greenberg 2008). In parallel, these goals, in turn, influence the behavior of teachers in the classroom and at school in general (Kramarski and Michalsky 2009).

Teachers’ continuous professional development and learning is one of the key factors in improving the quality of schools (Jennings and Greenberg 2008; Kramarski and Michalsky 2009), but it is also one of the most important mediators in making educational policy relevant to teachers and teaching practice more effective (Desimone et al. 2006). For these reasons, professional development is essential in education (Killion 2010). Researchers highlight different skills that educators must accomplish. These include knowledge of teaching strategies, classroom management, and learning environment (Desimone et al. 2006). In addition, it is suggested that teachers should be aware of their students and their learning potential, and undoubtedly, they should delve deeper into the subject they teach and understand and apply the curriculum project in the classroom. They should also have the knowledge and required skills to be able to work with pupils from different social, cultural, and linguistic backgrounds (Killion 2010). Prior research has substantiated that the effective transformation or application of tacit knowledge in the classroom may improve students’ ability to comprehend and solve problems, thereby enhancing their sustainable well-being (Killion 2010; Chamidy et al. 2020). Furthermore, improving teachers’ training is an essential parameter for enhancing the quality and effectiveness of education. Teachers’ professional development is a key factor in the quality of educational work as it has a significant positive impact on teachers’ beliefs, behavior, and practices (Chamidy et al. 2020).

In parallel, teachers who have not mastered such competence are likely to struggle to provide a sound teaching and learning environment and may feel emotionally exhausted (Tan et al. 2019). The social and interpersonal aspect of teaching is important because a teacher’s ability to relate to students goes far beyond issues of management and control. Cultivating and creating positive relationships with students has consistently been shown to enhance student motivation and engagement.

It is essential to mention that a teacher with a high sense of self-efficacy can motivate their students to maintain active participation in the classroom and strengthen their efforts to manage the difficulties they face, thus enhancing the self-efficacy of the students themselves. Conversely, teachers with a low sense of self-efficacy spend more time on non-academic subjects, and more easily abandon students who do not quickly show positive results. Finally, teachers who have doubts about their competence create educational environments that are likely to undermine students’ sense of effectiveness, as well as their cognitive development (Mohamed 2021).

### 1.3. Teachers’ Metacognitive Knowledge

Another important aspect that we must highlight is the relationship between metacognitive knowledge and tacit knowledge and the role they play in learning. A necessary condition for teaching students to be metacognitive is a pedagogical understanding of metacognition. Pedagogical understanding refers to teachers’ knowledge regarding effective instruction for helping students achieve a goal; in this case, becoming metacognitive. Metacognition is related to one’s awareness of one’s level and capabilities of thinking, as well as control over the activities one experiences. Therefore, it refers to the knowledge we have about our individual cognitive processes. It is associated with our ability to know exactly what we know and what we do not know (theoretical aspect of metacognition) and how to know it (practical aspect of metacognition) (Desimone et al. 2006; Killion 2010). The basic processes of metacognition are planning, self-control, and evaluation. Metacognition acquires the methodology and ability to design a targeted finding and production of the necessary information, is aware of the steps that contribute to the successful solution of a problem, and finally, evaluates the results and course of thought (Henry et al. 2011).

The way in which the individual shapes metacognitive knowledge about oneself as a cognitive being arises from information derived from the subjective experience of the individual about himself in relation to the work. This information, once the person is aware of it, is deposited in their memory as declarative knowledge, and consequently, can be used in relation to judgments about himself when it comes to facing a similar task (Tan et al. 2019).

The importance of conscious thought control is of great significance, especially in the field of education, as it allows individuals to better manage their cognitive abilities, and to identify weaknesses that can be corrected by constructing new cognitive skills. More specifically, metacognitive knowledge has direct consequences on educational practice, as it enables the teacher and their students to reflect on knowledge and its production and actively try to guide and regulate it.

The ability of a person to become aware of their thought processes gives them the ability to intervene, control, or correct them to achieve specific goals or learn in different educational settings (Eaude 2015; Stemler and Sternberg 2006).

### 1.4. The Present Study

The present paper attempts to investigate the pattern of relationships among the tacit knowledge (TK) of secondary school teachers, their professional development/growth, and their metacognitive knowledge about the difficulties concerning their teaching practices. The assessment of this pattern of relationships may be one important step toward understanding the components of skilled teaching in the Greek cultural context. The current paper is based on a larger research study that was carried out in the context of the doctoral dissertation of the second author about tacit knowledge as the theoretical framework for evaluating the effectiveness of high school teachers in Greece. The investigation of tacit knowledge as a framework of potential responses to the numerous social interactions that teachers encounter in their working environment could help to improve our understanding of teachers’ practices and enable insight into the nature, development, and use of teachers’ tacit knowledge.

The primary aim of the study, which is presented in the current paper, was the evaluation of the effect of metacognitive knowledge and professional development of high school teachers on the use of strategies stemming from their tacit knowledge. Additionally, a secondary aim of the current study was the evaluation of the effect of metacognitive knowledge on the professional development of high school teachers. The potential contribution of professional development/growth to the emergence of teachers’ tacit knowledge would facilitate an understanding of the conditions in which tacit knowledge develops. Research data have shown that the most favorable conditions for teachers’ professional learning are formed within the school context, as observation strengthens their perception so that they can use their professional judgment more effectively to shape more appropriate TK strategies (Eaude 2015). This has led to the hypothesis that the process of teachers’ professional learning could contribute to a potential acquisition or even decrease in the use of certain teachers’ TK strategies. For this reason, the present study expected (hypothesis 1) either positive or negative relations, depending on the type of TK strategy, between professional development/growth, especially social interaction, and the use of teachers’ tacit knowledge strategies. In addition, we hypothesized that the relations between teachers’ perceived difficulties, which constitute a part of their metacognitive knowledge regarding instruction, in situations concerning their teaching practices on the one hand and their tacit knowledge on the other were expected to be both positive and negative and either direct or indirect (via teachers’ professional development) (hypothesis 2), as the high metacognitive knowledge of different difficulties in handling specific social conditions is very likely to lead teachers to reflect on their TK strategies and, based on their level of professional development, to implement what they think is appropriate for them in the specific situation (Eaude 2015; Stemler et al. 2006).

## 2. Method

### 2.1. Participants

The participants of the study were Greek natives, who were recruited from 50 different schools of the islands of Creta and Cyclades, as well as the city of Thessaloniki in north Greece. The total sample of the present study was convenient and consisted of 279 secondary school teachers of both sexes (83 men (29.7%) and 196 women (70.3%)), between the ages of 30 and 59 years. Specifically, 40.1% (112) of the participants belonged to the age group of 30 to 39 years, 47.7% of the sample (133) belonged to the age group of 40–49 years, and 12.2% of the sample (34) belonged to the age group of 50–59 years. The participants represented all subject areas and varied in the amount of teaching experience, ranging from 1 to 19 years. In particular, 16 participants (5.7%) had 4 years of experience, 96 (34.4%) had 5–9 years of experience, 95 (34.1%) had 10–14 years of experience, and 72 (25.8%) had 15–19 years of teaching experience.

### 2.2. Measures

#### 2.2.1. Tacit Knowledge Inventory for High School Teachers (TKI-HS)

The TKI-HS is an inventory that Stemler et al. (2006) designed for the assessment of high school teachers’ tacit knowledge strategies, which they tend to use during their interaction with people in their professional (high school) environment. The TKI-HS is a situational judgment test that presents 11 written hypothetical scenarios of problem situations that are typically encountered by secondary school teachers in school life. Each of the challenges described in the scenarios is related to one of the following four categories: (i) relating to students; (ii) relating to other teachers of similar status; (iii) relating to senior teaching staff in management roles (supervisors); or (iv) relating to parents. 

Each hypothetical scenario provides seven (7) response options stating what the principal actor in the scenario “should do”. Response options were designed from the outset to fit one of seven (7) different response options, following TK types of strategies, that can be applied to a wide range of conditions: (1) “Avoid” (ignore the problem or avoid getting involved in the situation described); (2) “Comply” (do whatever I am asked to, by whoever may ask it); (3) “Confer” (discuss the problem with the person who causes it, attempting to provide a reasonable explanation of my point of view); (4) “Consult” (turn to someone else for advice or ask for mediation to resolve the problem); (5) “Delegate” (delegate responsibility for resolving the problem onto someone else); (6) “Legislate” (introduce new policies to deal with future situations, similar to the one described); and (7) “Retaliate” (without intention to improve the problem situation, react as an act of revenge toward the way you perceive the aggressor acted toward you) (Grigorenko et al. 2006; Stemler et al. 2006). 

Participants were asked to rate the appropriateness of each of the seven (7) types of TK strategies to be provided as a resolution response option to the practical problem described in each of the 11 hypothetical scenarios, using a 1–5 point Likert scale (1 = strongly disagree, 5 = strongly agree). For the translation of the TKI-HS inventory in Greek, we followed the International Test Commission (ITC) guidelines (www.intestcom.org accessed on 30 May 2023). A back-translation procedure was also followed for the elimination of any inconsistencies that would disrupt the accuracy of the results (Foutsitzi 2017).

The structure of the TKI-HS inventory, designed by Stemler et al. (2006), suggests that the 77 items of the 11 hypothetical scenarios’ inventories are loading, per eleven, on one of the seven types of TK strategies. Foutsitzi (2017) tested the aforementioned factor structure of the Greek version of the Tacit Knowledge Inventory for High School Teachers (TKI-HS) (Stemler and Sternberg 2006) by conducting confirmatory factor analyses (CFA) on the data collected from the 11 items, corresponding to each of the seven types of TK strategies, to determine their level of fit with the model suggested by the TKI-HS inventors. For the Greek version of TKI-HS, CFA verified the single-factor structure of each of the seven TK strategies but showed that the single-factor structure of the “Delegate” strategy, the “Confer” strategy, and the “Consult” strategy, which was finally confirmed, comprises 10 items; the single-factor structure of the “Avoid” strategy, the “Retaliate” strategy, and the “Comply” strategy, which was finally confirmed, comprises 7 items; while the single-factor structure of the “Legislate” strategy, which was finally confirmed, comprises 5 items (Foutsitzi 2017). Means and standard deviations of the participants’ TK strategies used according to age band, gender, and years of experience are depicted in Table 1. 

Furthermore, exploratory factor analysis (EFA) was applied by Foutsitzi et al. (2020) to all seven types of TKI-HS strategies in order for us to be able to identify the number of their organization’s underlying factors. The use of EFA revealed that the “avoid”, “delegate”, and “retaliate” TK strategies were loading onto a first factor that is labeled “indifference and avoidance”. The “confer” and “legislate” TK strategies were found to be loaded onto a second factor that is labeled “active response”, while the “comply” and “consult” strategies were found to be loaded onto the third factor that is labeled “Passive attitude”.

Taking into account that CFA and EFA verified/revealed slightly differentiated factor structures for both the total Greek version of the TKI-HS inventory and its scales compared to those suggested by its constructors, Cronbach’s alpha internal consistency estimates obtained in the present study for the aforementioned inventory and its factors/scales (see Foutsitzi et al. 2020) were as follows: *α* = 0.71, for the total TKI-HS inventory (number of items: 56); *α* = 0.72 for the factor that is labeled “indifference and avoidance” (number of items: 24); *α* = 0.62 for the factor that is labeled “active response” (number of items: 15); and *α* = 0.69 for the factor that is labeled “passive attitude” (number of items: 17). Although the aforementioned Cronbach’s alpha internal consistency estimates obtained in the present study are marginally acceptable (acceptable and questionable), they were also found to be comparable to those obtained by Elliott et al. (2011). In their study, Cronbach’s *α* coefficients for the “Good Response” scale (number of response options on the scale: 18) of the TKI-HS strategies ranged from 0.64 to 0.76, and Cronbach’s *α* coefficients for the “Bad Response” scale (number of response options on scale: 19) of the TKI-HS strategies ranged from 0.65 to 0.71.

Additionally, in the present study, the reliability coefficients for the seven TKI strategies were found to range from 0.40 to 0.80, and except for Cronbach’s alpha, we also took into account the following reliability coefficients: (a) greatest lower-bound reliability (GLB reliability); (b) Bentler’s dimension-free lower-bound reliability; and (c) Shapiro’s lower-bound reliability. 

The specifics are as follows:

(1) for the “Avoid” strategy (number of items: 7):
  Cronbach’s *α* = 0.51, GLB reliability = 0.61, Bentler’s dimension-free lower-bound reliability = 0.61, and Shapiro’s lower-bound reliability = 0.67;
(2) for the “Delegate” strategy (number of items: 10):
  Cronbach’s *α* = 0.66, GLB reliability = 0.75, Bentler’s dimension-free lower-bound reliability = 0.75, and Shapiro’s lower-bound reliability = 0.80;
(3) for the “Retaliate” strategy (number of items: 7):
  Cronbach’s *α* = 0.46, GLB reliability = 0.55, Bentler’s dimension-free lower-bound reliability = 0.55, and Shapiro’s lower-bound reliability = 0.57;
(4) for the “Confer” strategy (number of items: 10):
  Cronbach’s *α* = 0.55, GLB reliability = 0.68, Bentler’s dimension-free lower-bound reliability = 0.68, and Shapiro’s lower-bound reliability = 0.26;
(5) for the “Legislate” strategy (number of items: 5):
  Cronbach’s *α* = 0.40, GLB reliability = 0.46, Bentler’s dimension-free lower-bound reliability = 0.46, and Shapiro’s lower-bound reliability = 0.47,
(6) for the “Comply” strategy (number of items: 7):
  Cronbach’s *α* = 0.54, GLB reliability = 0.65, Bentler’s dimension-free lower-bound reliability = 0.65, and Shapiro’s lower-bound reliability = 0.6;
(7) for the “Consult” strategy (number of items: 10):
  Cronbach’s *α* = 0.62, GLB reliability = 0.70, Bentler’s dimension-free lower-bound reliability = 0.70(Shapiro’s lower-bound reliability could not be calculated because of failure to converge in 500 iterations).

Since we have not identified any information in the current paper on the proposed organization and internal consistency reliability of the seven types of TKI-HS strategies in the context of the literature review, the low-reliability coefficients of the aforementioned scales are perhaps due to their small number of items. It is noted that the “Legislate” TK strategy was not included further in the present study since all its reliability coefficients were unacceptable.

#### 2.2.2. Life Challenges Teachers Inventory

For the evaluation of the professional development/growth of teachers, the “Life Challenges Teachers Inventory”, a self-report questionnaire, was applied. The current questionnaire was developed by Iluz et al. (2012) and is a transfer of the application of the three-factor theoretical framework of the Definition and Selection of Competencies Project (DeSeCo) (OECD 1997, 2002) to teachers’ professional context.

The inventory consists of 35 items originally formulated in the Hebrew language, which were translated by its constructors into English. Participants were asked to provide some general information on how they responded to their educational tasks, indicating their degree of agreement or disagreement with the content of the questionnaire items (OECD 2002). Teachers’ answers are given in the form of a six-point Likert scale, where 1 stands for “strongly agree” and 6 stands for “strongly disagree”. 

Regarding the structure of the inventory, according to its constructors, its 35 items are loaded on three scales/factors. The first scale named “Using tools interactively” concerns the ability of individuals to effectively use a significant number of tools to achieve an effective interaction with both their physical and socio-cultural environment. The “Using tools interactively” scale consists of 12 items, which are loaded on three subscales: “Language”; “Knowledge”; and “Technology” subscales. The second scale, “Interacting in socially heterogeneous groups”, refers to the ability of the individual to communicate effectively with people from different professional and cultural backgrounds and to form relationships within different learning environments. It consists of nine items, which are loaded on three subscales: the “Relationships” subscale, the “Teamwork” subscale, and the “Resolving conflicts” subscale. The third scale, “Acting autonomously”, concerns the ability of the individual to act autonomously, take initiative, and motivate themself. It consists of 14 items, which are loaded on the “Independence” subscale, the “Initiative” subscale, and the “Motivation” subscale. For the translation of the “Life Challenges Teachers Inventory” in Greek, we followed the International Test Commission (ITC) guidelines (www.intestcom.org accessed on 30 May 2023). The back-translation procedure also followed the elimination of any inconsistencies that would disrupt the accuracy of the results (Foutsitzi 2017). 

Foutsitzi (2017) tested the abovementioned factor structure of the Greek version of the Life Challenges Teachers Inventory by conducting confirmatory factor analyses on the data collected from the 35 items, corresponding to each of the three scales, to determine their level of fit with the model suggested by the constructors of the “Life Challenges Teachers Inventory”. For the Greek version of the inventory, CFA verified a slightly differentiated structure than the one suggested for each scale by its constructors. In particular, the factor structure of the “Using tools interactively” scale, which was finally confirmed, comprises 11 items (Cronbach’s *α* = 0.83). Furthermore, the factor structure of the “Interacting in socially heterogeneous groups” scale, which was finally confirmed, comprises eight items (Cronbach’s *α* = 0.71), while the factor structure of the “Acting autonomously” scale, which was finally confirmed, comprises all the fourteen items (Cronbach’s *α* = 0.83), proposed by its inventors (Iluz et al. 2012).

It is noteworthy that although CFA verified a slightly differentiated factor structure than the one suggested for each scale by its constructors, Cronbach’s alpha internal consistency estimates, obtained in the present study, were also found to be acceptable and comparable to those obtained by Iluz et al. (2012): *α* = 0.83 (Iluz et al. 2012: *α* = 0.89) for the “Using tools interactively” scale; *α* = 0.71 (Iluz et al. 2012: *α* = 0.78) for “Interacting in socially heterogeneous groups”; and *α* = 0.83 (Iluz et al. 2012: *α* = 0.86) for “Acting autonomously”.

#### 2.2.3. Teachers Metacognitive Knowledge Inventory

The “Teachers Metacognitive Knowledge Inventory” is a self-report questionnaire, which was constructed by Foutsitzi (2017) that aims to investigate teachers’ metacognitive knowledge regarding the difficulties, shortcomings, and weaknesses that hinder their educational practices. The questionnaire contained 21 items, originally formulated in the Greek language. The items reflect the difficulties that teachers face daily and over time in their immediate and wider work environment. Participants were asked to rate the extent to which they comprehend that they are experiencing difficulties. The teachers’ answers were given in the form of a 6-point Likert scale, where 1 stands for “strongly disagree” and 6 stands for “strongly agree”. Regarding the structure of the inventory, according to its constructor (Foutsitzi 2017), after the conduction of both exploratory and confirmatory factor analyses, its 21 items were found to be loaded on three scales/factors: the first scale named “Difficulty in Classroom Management” comprises 10 items (Cronbach’s *α* = 0.84); the second scale named “Difficulty in the Use of Modern Methods and Technologies” comprises 5 items (Cronbach’s *α* = 0.87); and the third scale named “Difficulty in Cooperation and Address to Infrastructure Deficiencies” comprises 6 items (Cronbach’s *α* = 0.65). 

### 2.3. Procedure

Information about the research and its purpose was given to participants before administering the measures. All participants were informed that their participation was voluntary and reassured about the anonymity of the results. Written informed consent was obtained for all the participants who were subsequently asked to fill out an individual demographics form. The TKI-HS Inventory, the Life Challenges Teachers Inventory, and the Teachers Metacognitive Knowledge Inventory were administered by the second author to the participants, either individually or in groups of 3–4 persons, in a quiet school room where each teacher worked to minimize the presence of any disruptions and disturbances. Data collection was carried out before the COVID-19 pandemic period. The duration of the examination was approximately 30–45 min.

### 2.4. Statistical Analysis

To investigate the aims of the present study, path analysis was conducted. Considering that path analysis, a structural equation modeling (SEM) technique for analyzing structural models with observed variables, is adequate for examining causal relationships among multiple constructs measured using summated scales (Bentler 2006; Hu and Bentler 1999), we proceeded with this analysis. Specifically, to examine the relationships between metacognitive knowledge, professional growth, and tacit knowledge, path analysis with manifest variables was computed. Because of the relatively small sample size, the analysis was not run at the item level (observed/measured variables). Instead, the covariance matrix was based on total scores (latent variables) of metacognitive knowledge (“Difficulty in Classroom Management”; “Difficulty in the Use of Modern Methods and Technologies”; “Difficulty in Cooperation and Address to Infrastructure Deficiencies”), of professional development/growth (“Using tools interactively”; “Interacting in socially heterogeneous groups”; “Acting autonomously”), and of the six (6) different TK types of strategies (“Avoid”; “Comply”; “Confer”; “Consult”, “Delegate”; and “Retaliate”). The “Legislate” TK strategy was not included in the path analysis because all its reliability coefficients were unacceptable. The indicators of the TK types of strategies and professional development were defined as endogenous variables. The three metacognitive knowledge indicators were defined as exogenous variables.

## 3. Results

**Figure 1:**
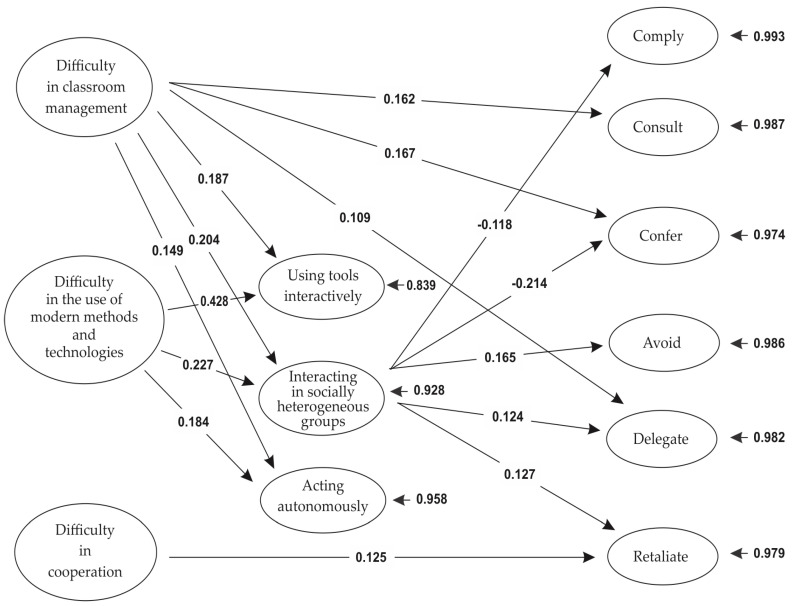
Schematic representation of the final path model displaying the network of relationships among variables of metacognitive knowledge, professional development, and tacit knowledge strategies.

Path analysis was conducted in EQS 6.1. and performed on a covariance matrix using the maximum-likelihood estimation procedure (Bentler 2006). Various models were applied, incorporating the suggested modifications resulting from the examination of the residuals and Lagrange multiplier and Wald tests’ performance each time. The final model (representing the network of statistical associations between metacognitive knowledge and professional development variables with tacit knowledge variables) is presented in Figure 1. The model had an excellent fit to the data of the present research [χ^2^ (34) = 32.62, *p* = .53, CFI = 1.00, SRMR = 0.04, RMSEA = 0.00 (CI90% 0.00–0.04)]. This model includes only statistically significant variables, statistically significant loadings, and statistically significant covariance between the abovementioned variables. The depiction of the relationships of all variables is presented in Figure 1.

### 3.1. Effect of Teachers’ Professional Development/Growth on Tacit Knowledge Strategies

Of the three components of teachers’ professional development, only deficiencies in interacting in socially heterogeneous groups were found to influence almost all ΤΚ strategies, which reflect teachers’ practical intelligence. More specifically, the reduced ability of the teacher to communicate effectively with people from different professional and cultural backgrounds and to form relationships within different learning environments was found to have a negative effect, to a moderate extent (percentage of explained variance of about 4.58%), on the use of the “Confer” strategy. Obviously, the less cooperative a teacher is, and the less effective communication they have, the less they tend to choose the strategy “Confer” in the context of their social interactions in the school environment, according to the above finding. Furthermore, the deficiencies in interacting in socially heterogeneous groups were also found to have a negative effect, to a lesser extent (percentage of explained variance of about 1.39%), on the use of the TK “Comply” strategy. According to this finding, it is possible that when teachers can collaborate and communicate effectively with people in their workplace, they seem to adopt the “Comply” strategy more often in their professional relationships. 

Finally, the deficiencies of interacting in socially heterogeneous groups were found to have a positive effect, to a small extent, on the use of the TK strategies “Avoid” (percentage of explained variance of about 2.72%), “Delegate” (percentage of explained variance of about 1.54%), and “Retaliate” (percentage of explained variance of about 1.62%). According to this finding, the less cooperative a teacher is, the more they tend to choose, in the context of their social interactions, strategies that do not involve the personal handling of problems that arise. By adopting the “Avoid” strategy, the teacher seems to overlook the problem and avoids any participation in its solution. The adoption of the “Delegate” strategy is the transfer of responsibility for solving the problem to someone else, while the “Retaliate” strategy is the adoption by the teacher of an attitude like that used by the person with whom they are in conflict. At this point, it should be noted that the above findings highlight the relationship between teachers’ professional development and tacit knowledge, and partially confirm Hypothesis 1, as they demonstrate the existence of both positive and negative relationships between teachers’ deficiencies in professional development in interacting with socially heterogeneous groups and their use of certain TK strategies. Figure 1 depicts a schematic representation of the network of relationships among variables of professional development and tacit knowledge strategies.

### 3.2. Effect of Teachers’ Metacognitive Knowledge on Tacit Knowledge Strategies

Metacognitive knowledge regarding the difficulties in classroom management was found to have a direct and positive, to a small extent, effect on the use of TK strategies “Consult” (percentage of explained variance of about 2.62%), “Confer” (percentage of explained variance of about 2.79%), and “Delegate” (percentage of explained variance of about 1.19%). The choice of the “Consult” strategy indicates the teacher’s intention to seek someone else’s advice or mediation to solve the problem they are facing. The choice of the “Confer” strategy, under the same conditions, dictates a more active mediation of the teacher to solve the problem, as this strategy implies the personal intervention of the teacher to handle and solve the problem. Finally, the choice of the “Delegate” strategy consists of shifting the responsibility for solving the problem from the teacher himself to someone else, indicating an inability to manage the situation themself. These findings suggest that the more difficulty a teacher feels in maintaining discipline and control in the classroom, the more they directly choose to use the TK “Consult”, “Confer”, and “Delegate” strategies. 

Furthermore, the difficulty in classroom management was found to have an indirect positive effect on the use of the TK “Delegate” strategy, as well as on the TK “Avoid” and “Retaliate” strategies, through its inhibitory effect on one of the professional development components, namely interaction in socially heterogeneous groups (see Figure 1). Furthermore, path analysis indicated an indirect negative effect of the metacognitive knowledge of difficulty in classroom management through the reduced social interaction in heterogeneous groups on the use of TK “Comply” and “Confer” strategies. 

Metacognitive knowledge about the difficulty in the use of modern methods and technologies was not found to directly affect the use of any of the seven strategies of tacit knowledge. However, it was observed that, similarly to the difficulty in classroom management, it indirectly and negatively affects the use of the “Comply” and “Confer” TK strategies and indirectly and positively affected the use of the “Avoid”, “Delegate”, and “Retaliate” TK strategies through its effect on the reduction in interacting in socially heterogeneous groups (professional development variable). Figure 1 depicts a schematic representation of the network of relationships among variables of metacognitive knowledge, professional development, and tacit knowledge strategies. 

On the contrary, the difficulty in cooperation and addressing infrastructure deficiencies was found to have a direct positive effect, to a small extent (percentage of explained variance of about 1.56%), exclusively on the use of the TK “Retaliate” strategy. This finding reveals that the more a teacher finds it difficult to work with the people and to deal with infrastructure deficiencies in their working environment, the more often they tend to adopt the TK “Retaliate” strategy to manage specific social situations that arise in the school context. Finally, it is essential to mention that the difficulty in cooperation and addressing infrastructure deficiencies was not found to have an indirect effect on any of the seven tacit knowledge strategies. 

At this point, it should be noted that all the above findings largely confirm hypothesis 2 and demonstrate the existence of either direct or indirect (positive and negative) relationships between teachers’ metacognitive knowledge and their tacit knowledge.

## 4. Discussion

The results presented above refer to the paths and the extent to which secondary school teachers’ metacognitive knowledge, regarding their difficulty in (i) classroom management, (ii) use of modern methods and technologies, and (iii) cooperation and addressing infrastructure deficiencies, as well as their deficiencies in issues of professional development/growth (“Using tools interactively”; “Interacting in socially heterogeneous groups”; and “Acting autonomously”) could affect the type of strategies, stemming from their tacit knowledge, that they tend to use during their interaction with people in their professional (high school) environment. 

### 4.1. Relationships between Teachers’ Professional Development and Tacit Knowledge

More specifically, regarding the effect of the deficiencies of professional development on the use of the TK strategies, it was found that deficits in interacting (cooperating and communicative effectively) “In socially heterogeneous groups” push teachers to choose strategies that relieve them of responsibility of taking action to solve problems that arise at school. The adoption of the strategies “Avoid”, “Delegate”, and “Retaliate” by the teacher highlights an almost indifferent attitude on their part toward the problem that has arisen, revealing an inability to personally manage the situation that arose in the context of their work environment. The lack of capacity for cooperation, effective communication, and effective problem solving could be interpreted as a sign of limited social intelligence (Krüger et al. 2007; Scully et al. 2019; Stemler et al. 2018). Due to its partial overlying with practical intelligence the ability, that is, adaptation to social conditions (Ash 2004; Emmerling and Boyatzis 2012; Mulaik and Millsap 2000), and the choice of the appropriate strategy for handling these social conditions, it may also result in limited practical intelligence, thus interpreting the choice of the above strategies by the teacher, which are unauthorized to address the situation. On the contrary, the greater the ability of the teacher in the field of cooperation and communication in socially heterogeneous groups, the more they were found to tend to choose the TK strategies “Confer” and “Comply”.

These strategies indicate an active willingness of the teacher to participate in handling the problem through the effortless exchange of messages and the interaction of those involved in the social treaty addressed. The ability to communicate effectively includes social skills, such as the ability to create, maintain, and manage personal relationships with others (Emmerling and Boyatzis 2012) and understand the dynamics of a conversation and negotiate, as well as the ability to manage and resolve problems and conflicts (Iluz et al. 2012). The more proficient a teacher feels in these skills, the more direct and active their participation is expected to be in resolving conflicts and problems, which are an integral part of human relations. The adoption of the strategy “Confer” by the teacher interprets the intention of an active effort on their part to solve the problem through the presentation of logical arguments and justification of their choices and positions (Emmerling and Boyatzis 2012). 

### 4.2. Relationships between Teachers’ Metacognitive Knowledge and Tacit Knowledge via Their Professional Development

Path analysis revealed that teachers’ metacognitive knowledge regarding the difficulties they face in issues such as classroom management and the use of modern methods and technologies inhibits their professional development. In fact, the findings of the path analysis revealed that metacognitive knowledge regarding the difficulty in classroom management enhances the shortages of professional development in “Using tools interactively”, “Interacting in socially heterogeneous groups”, and “Acting autonomously”. Classroom management is an important educational process that affects the professional functionality of a teacher (Kramarski and Michalsky 2010). The teachers’ perceived difficulty in this field prevents them from acting independently and on their own initiative (Woolfolk Hoy and Burke-Spero 2005) limits their work satisfaction, undermines their careers, and prevents them from participating in professional learning. These processes would aid in becoming familiar with alternative teaching methods and their adoption of a significant number of tools that could facilitate the teachers’ effective interaction with the physical and socio-cultural environment and make them able to cooperate with the members of the educational community and take initiative in their teaching practice. Similarly, teachers’ metacognitive knowledge regarding the challenges that they perceive with using modern methods and technologies limits or completely eliminates their autonomy in actions and initiatives, as well as their ability to form cooperative relationships and engage in socially heterogeneous groups within their work environment. The fear of failure and losing control that instructors may experience because of using current methods and technologies is likely to increase, preventing them from engaging in professional learning processes (Freiberg et al. 2013; Grigorenko et al. 2004; Hobson et al. 2008). 

In addition, metacognitive knowledge regarding the difficulty in classroom management, as well as the difficulty in the use of modern methods and technologies, were found to indirectly and positively affect the use of the TK “Delegate” strategy, as well as the “Avoid” and “Retaliate” strategies through their inhibitory effect on the component of professional development, namely “Interaction in socially heterogeneous groups”. According to these findings, communication between teachers is limited by their inability to work together effectively due to the challenges of regulating the classroom and utilizing contemporary techniques and technologies. As a result, teachers frequently select less participatory techniques, such as the ones indicated above (“Delegate”, “Avoid”, and “Retaliate”). 

Furthermore, the indirect negative effect of metacognitive knowledge of difficulty in classroom management and in the use of modern methods and technologies, through social interaction in heterogeneous groups, on the use of the TK “Comply” and “Confer” strategies were also found, demonstrating the vital importance of effective communication and cooperation for the selection of appropriate strategies that require the active participation of the teacher in regulating the problems that arise.

### 4.3. Relationships between Teachers’ Metacognitive Knowledge and Tacit Knowledge

Specifically, it was observed that the difficulty faced by teachers in matters of classroom management, one of the most important problems that teachers are pointed out to face in their teaching practice, pushes them to directly choose TK strategies such as “Consult” and “Delegate”, which are rather passive and indirect and involve transferring the responsibility for the solution of the problem to someone else. Teachers who reported that they were facing classroom management difficulties tended to choose to tackle the problems that were more strategically dictated, either through the mediation of someone else or by shifting the responsibility and avoiding the immediate and active intervention of the teacher themself. The choice of TK strategies “Consult” and “Delegate” interprets the pursuit of teachers to be the disposal of the obligation to make unpleasant decisions and the negative feelings of anxiety they are experiencing because of the inability to maintain control in the classroom. It was also found that difficulty in classroom management can also push the teachers to choose the TK “Confer” strategy, which dictates a more active and personal handling of the problem on the part of the teacher. This choice was interpreted as a possible result of a generally interventionist attitude adopted by the teacher in their teaching practice. The selection of the strategy “Consult” by teachers who said they were facing classroom management difficulties reflects the effect of metacognitive knowledge of this kind of difficulty on a strategy that points to an immediate and active intervention by the teacher to solve the problem in the classroom. This finding can be interpreted if we consider the fact that teachers’ decisions on classroom management are significantly influenced by their attitudes and beliefs on these issues (Beach and Pearson 1998; Grigorenko et al. 2004). Consequently, a teacher who has adopted an interventionist approach to class management issues is likely to choose strategies to allow them to exercise a high degree of control over their class activities (Garet et al. 2001; Jones and Jones 2012; Witcher et al. 2001). 

The difficulty faced by the teacher in matters of cooperation and addressing infrastructure deficiencies was found to lead the teacher to directly choose the TK strategy “Retaliate”, which signals an immediate and impulsive reaction to the situation being faced, highlighting deficiencies in social and practical skills, which would allow the selection and effective implementation of an appropriate strategy for handling the problem that has arisen. In fact, the choice of this TK strategy highlights a reaction like the one the teacher receives from the person with whom they are in confrontation, which may intensify the confrontation. The difficulty in cooperation is often the result of an inability to comprehend and understand the attitudes, actions, and reactions of others, and it hinders coping with the unpleasant situation (Emmerling and Boyatzis 2012; Jones and Jones 2012; Scully et al. 2019). 

### 4.4. Conclusions

A limitation of the current study is the administration of self-report inventories, which reduces the objectivity of the measurement (Adnan and Anwar 2020). Teachers’ expectations for their professional image and self-esteem, their willingness to disclose certain beliefs and emotions, or even the socially desired outcome may have influenced their perceptions, and consequently, their responses. Another limitation is the slight representation of participating teachers with less than five years of teaching experience. 

Furthermore, according to its creators, the TKI-HS instrument (as a situational judgment test) would be more well suited to Q-type factor analysis or latent profile analysis (LPA), which is a categorical latent variable approach that focuses on identifying latent subpopulations within a population based on a certain set of variables. Therefore, another limitation of the present study is the effect of the use of traditional R-type factor analyses as a way to test the factor structure of the TKI-HS inventory. One pitfall of the R-type factor analysis approach is the fact interaction between the TKI-HS strategy and the situation wherein it is sometimes the “wrong” approach to comply, sometimes the “right” approach, and sometimes in the middle. When that is not taken into account, it would likely fail to reverse-code an item on a survey. Consequently, when one runs an R-type factor analysis in such circumstances, the internal consistency reliability coefficients are poor. Additionally, though researchers often provide information about the internal consistency reliability of situational judgment tests (SJTs), this estimate is known to be unsuitable for SJTs because they are heterogeneous tests, and a better estimate for the reliability of SJTs may be retest reliability as the authors of a recently published meta-analysis propose (Harenbrock et al. 2023). As a result, a future study would be useful for the Greek version of TKI-HS to be analyzed with latent profile analysis (LPA) and its retest reliability to also be estimated in the population of Greek HS teachers. 

A final limitation of the current study is the fact that it was designed and conducted before the COVID-19 pandemic period. Education worldwide has been greatly affected by the COVID-19 outbreak. Schools chose to utilize conferencing software to deliver online lessons on an enormous scale in response to the pandemic to return things to normal (Adnan and Anwar 2020). As a result, it would be very interesting to evaluate the methods that teachers developed during the COVID-19 period regarding tacit knowledge conversion techniques for online learning, as well as to assess the impact of the approach on how well students internalize their tacit knowledge to satisfy the demands of online teaching and the restriction of teacher–student interactions because of online learning.

However, under the aegis of our research findings, this work could be seen as a preliminary attempt to evaluate the relationships among teachers’ tacit knowledge, professional growth, and metacognitive knowledge. The current findings show “Difficulty in Classroom Management” and “Interacting in socially heterogeneous groups” as the main and significant factors influencing almost all TK strategies, while “Difficulty in the Use of Modern Methods and Technologies” also had an indirect effect via “Interacting in socially heterogeneous groups” on TK strategies. Although more research is also needed to further replicate our current findings, the results of this study, with the size of the sample used, can lead to important practical implications. The practical implications of the research findings concern their exploitation in the training of teachers, contributing to the creation of conditions that could contribute to the strengthening of their effectiveness. As the research findings revealed the important role of relationships and communication among the members of the educational community, the need to organize teachers’ professional development programs, which will focus on the improvement of their interaction in socially heterogeneous groups, is of vital importance for the enhancement of their tacit knowledge. 

## Figures and Tables

**Table 1:** Means and standard deviations of tacit knowledge strategies according to age, gender, and years of experience of the total sample of participants.

Tacit Knowledge Strategies	Age			Gender		Total	Years of Experience			
	29–39	40–49	50–59	Men	Women		0–4	5–9	10–14	15–19
**AVOID**	15.071	14.736	15.411	14.478	15.112	14.953	14.435	14.760	15.221	14.972
	(2.967)	(3.190)	(2.986)	(3.104)	(3.058)	(3.076)	(2.529)	(3.127)	(3.206)	(2.964)
										
**COMPLY**	21.901	20.909	21.147	20.867	21.535	21.336	24.625	21.583	20.842	20.930
	(3.581)	(3.401)	(2.652)	(3.431)	(3.398)	(3.416)	(3.422)	(3.481)	(3.525)	(2.744)
										
**CONFER**	39.410	39.533	38.588	38.939	39.551	39.369	39.250	39.354	39.315	39.486
	(3.294)	(3.673)	(3.939)	(3.667)	(3.507)	(3.559)	(4.281)	(3.314)	(3.591)	(3.734)
										
**CONSULT**	34.794	33.864	33.147	34.686	33.923	34.150	35.000	34.770	34.052	33.263
	(4.047)	(4.719)	(3.955)	(4.666)	(4.264)	(4.393)	(4.305)	(4.524)	(4.367)	(4.185)
										
**DELEGATE**	21.401	21.082	21.852	21.819	21.086	21.304	19.500	21.114	21.768	21.342
	(4.155)	(4.214)	(4.226)	(4.513)	4.090)	(4.226)	(3.741)	(4.177)	(4.494)	(3.975)
										
**LEGISLATE**	18.410	18.601	17.705	18.337	18.449	18.415	19.312	18.520	18.252	18.291
	(2.260)	(2.222)	(2.236)	(2.359)	(2.205)	(2.248)	(2.750)	(2.200)	(2.143)	(2.322)
										
**RETALIATE**	16.694	17.691	18.117	18.253	17.112	17.451	15.937	17.364	17.326	18.069
	(3.366)	(3.321)	(3.300)	(3.645)	(3.168)	(3.351)	(2.839)	(3.586)	(3.394)	(3.351)

## Data Availability

Not applicable.
